# Capsular attachment of the subregions of rotator cuff muscles

**DOI:** 10.1007/s00276-019-02288-7

**Published:** 2019-07-11

**Authors:** Takuma Yuri, Hiroto Kobayashi, Yuta Takano, Saori Yoshida, Akira Naito, Hiromi Fujii, Yoshiro Kiyoshige

**Affiliations:** 1 Graduate School of Health Sciences, Yamagata Prefectural University of Health Sciences, 260 Kamiyanagi, Yamagata, 990-2212 Japan; 2 Research Fellow of Japan Society for the Promotion of Science, Tokyo, Japan; 3 Department of Anatomy and Structural Science, Faculty of Medicine, Yamagata University, 2-2-2 Iida-nishi, Yamagata, 990-9585 Japan

**Keywords:** Capsular attachment, Rotator cuff, Supraspinatus, Infraspinatus, Subregion

## Abstract

**Purpose:**

This study aimed to morphologically and histologically investigate the relationship between deep subregions of the rotator cuff muscle and shoulder joint capsule as well as the relationship between the rotator cuff tendon or capsule and bony insertion.

**Methods:**

We examined 13 shoulders of embalmed cadavers and measured the capsular attachments and footprints macroscopically. We also histologically examined the fibres in three shoulders.

**Results:**

Loose attachment, which was less tight with spaced connective tissue, and firm attachment, which was tight with dense connective tissue, were found under the surface of the supraspinatus and infraspinatus. The anterior-deep and posterior-deep subregions of the supraspinatus and the middle partition and inferior partition of the infraspinatus formed firm attachments to the capsule. The mean areas of firm attachment for the anterior-deep subregion, posterior-deep subregion and middle partition were 118.8 mm^2^, 267.8 mm^2^ and 399.3 mm^2^, respectively, while the area of the inferior partition was small. The transverse fibres were located just lateral to the medial edge of the firm attachment area. The thick capsule had a substantial footprint. Both tendon fibres and the capsule inserted into the superior and middle facets through the attachment fibrocartilage.

**Conclusions:**

The posterior-deep subregion of the supraspinatus and middle partition of the infraspinatus evenly occupied the capsular attachment area. The transverse fibres were located just lateral to the medial edge of the firm attachment area, and the thick capsule had a substantial footprint. Both tendon fibres and the capsule inserted into the superior and middle facets through the attachment fibrocartilage.

## Introduction

Many investigators have mentioned the relationship between the rotator cuff tendon and gleno-humeral joint capsule [10, 12, 16] since Clark precisely studied it [5, 6]. According to Clark, the supraspinatus and infraspinatus have loose and firm attachments to the gleno-humeral joint capsule; Clark also suggested that the loose attachment retracts redundant capsule tissue, while the firm attachment distributes some of the tension generated by the rotator cuff muscles into the capsule [5]. They also found that the capsule was thick where it was most firmly attached to the cuff tendons, adjacent to their insertion into the tuberosities [5]. Nimura et al. reported that the articular capsule inserts into the greater tuberosity with a thicker footprint than previously thought [15]. Burkhart et al. found the semi-circular thickness, naming it a rotator cable and the crescent configuration distal to the rotator cable a rotator crescent [4]. Burkhart hypothesized that the rotator cable has a stress transfer function that transmits the tensions generated by the rotator cuff into the humerus through each end of the cable’s span; the authors named this idea the suspension bridge theory [3]. These results suggest that the gleno-humeral joint capsule and rotator cable contribute to joint movement.

Recently, the supraspinatus and infraspinatus were considered more complex than previously thought. For example, Kim et al. divided the supraspinatus into six subregions: the anterior-superficial, anterior-middle, anterior-deep, posterior-superficial, posterior-middle and posterior-deep subregions [10]. In the infraspinatus, Fabrizio and Clemente as well as Bacle et al. independently divided the infraspinatus muscle into three partitions: the superior, middle, and inferior partitions [1, 7]. Both supraspinatus subregions and infraspinatus partitions are supported by intra-muscular innervations [7, 9]. Yuri et al. and Kuwahara et al. demonstrated that these subregions are functionally distinct [11, 18] and that the posterior-deep subregion and middle partition have a similar function in external rotation [11]. These observations suggest that the posterior-deep subregion and middle partition independently contribute to joint movement. However, the relationship between these subregions and the gleno-humeral joint capsule or rotator cable remains unknown. Thus, this study aimed to morphologically and histologically investigate the relationship between deep subregions of the rotator cuff muscle and shoulder joint capsule as well as the relationship between the rotator cuff tendon or capsule and bony insertion. We hypothesized that the posterior-deep subregion of the supraspinatus and the middle partition of the infraspinatus formed capsular attachment since the posterior-deep subregion and middle partition have similar functions [11].

## Materials and methods

**Fig. 1:**
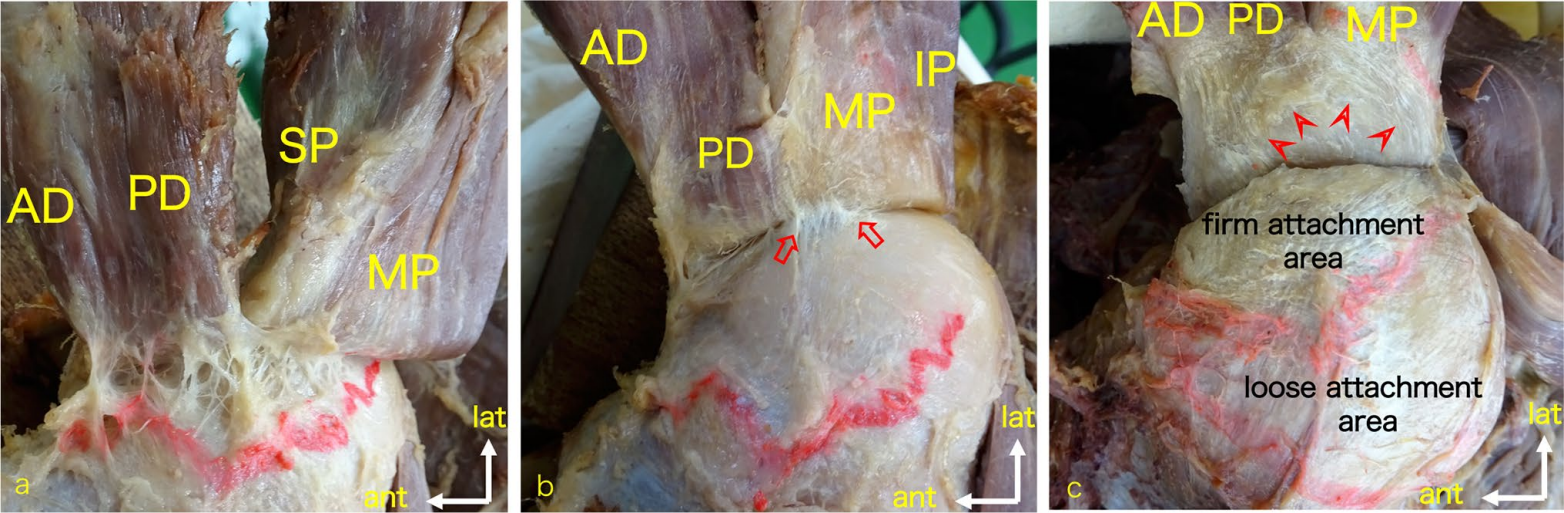
**a** Medial view of the loose attachment between the gleno-humeral joint capsule and the supraspinatus and infraspinatus subregions. The loose attachment, which was composed of less dense and well-spaced connective tissues, existed between the capsule and deeper muscle fibres of the anterior-deep subregion, posterior-deep subregion and middle partition. The medial margin of the loose attachment is marked with a red line. More than half of the anterior-deep subregion muscle fibres overlaid those of the posterior-deep subregion. The superior partition muscle fibres were located behind the middle partition. **b** Medial view of the firm attachment (arrows) between the capsule and the deep muscles and/or tendon fibres of the anterior-deep subregion, posterior-deep subregion and middle partition. The firm attachment was composed of more tight and dense connective tissue compared to the loose attachment. In this picture, the firm attachment was observed under tension only between the deeper surface of posterior-deep subregion and middle partition and the capsule because the firm attachment area was in an arch-like fashion, which was at the apex of the posterior-deep subregion and middle partition. **c** Medial view of the lateral end of the firm attachment. The medial margin of the firm attachment area is also marked with a red line. In an arch-like fashion, the transverse fibres (arrowheads) remained at the deeper surface of the rotator cuff tendon and just lateral to the mark when the firm attachment was sharply separated at the level between the tendon and the capsule. *AD* anterior-deep subregion, *PD* posterior-deep subregion, *IP* inferior partition, *MP* middle partition, *SP* superior partition, *ant* anterior, *lat* lateral (colour figure online)

**Fig. 2:**
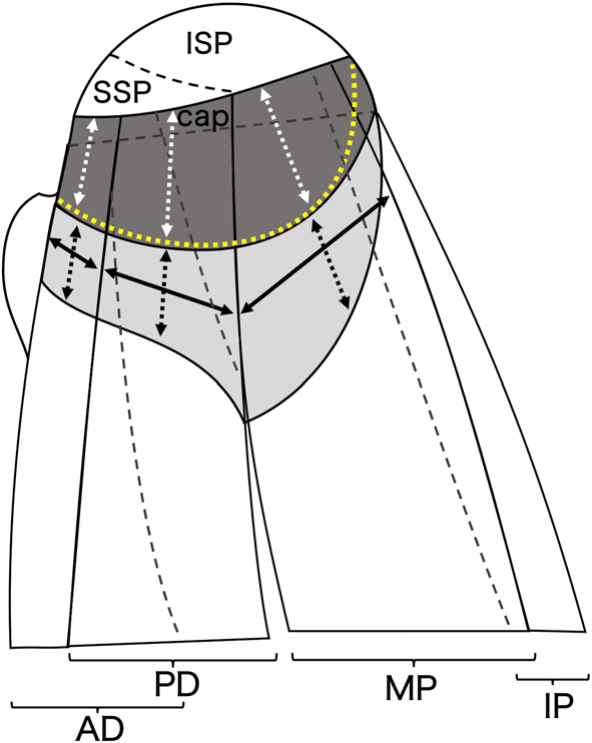
Superior schematic view of the deeper surface of subregions of the anterior-deep subregion, posterior-deep subregion, middle partition and inferior partition that attached the capsule and of the corresponding measurement methods. The dashed lines on the posterior-deep subregion delineated the overlap of the posterior-deep subregion by the anterior-deep subregion and middle partition. The dashed line on the middle partition delineates the overlap of the middle partition by the inferior partition. The loose and firm attachment areas are light and deep grey-shaded areas, respectively. The yellow dotted line delineates the transverse band, which is located just lateral to the medial margin of the firm attachment area (deep grey-shaded area). The dashed line on the greater tubercle delineates footprint areas of the supraspinatus and infraspinatus. The mediolateral lengths of the loose and firm attachments were measured at the midpoints of each subregion (dotted double head allows), and their anteroposterior widths were measured at the boundaries between the loose and firm attachments (double head allows). *AD* anterior-deep subregion, *PD* posterior-deep subregion, *IP* inferior partition, *MP* middle partition, *ISP* infraspinatus, *SSP* supraspinatus (colour figure online)

We first included 24 shoulders from 12 cadavers (10 males and 2 females; mean age at death, 80 years old) and assigned 13 for macroscopic and three for histological analyses after excluding eight shoulders with rotator cuff tears. The supraspinatus and infraspinatus were freed from their origins on the scapular fossa and reflected laterally. The dissection continued to the gleno-humeral joint capsule and the greater tubercle by separating the loose and firm attachments between the muscle or tendon fibres and the capsule. The loose and firm attachments were defined on the basis of the findings by Clark [5, 6]. Although the loose attachment areas were less tight with well-spaced connective tissue and were dissected bluntly, a scalpel was necessary to separate the firm attachment areas, which were tight and dense (Fig. 1). Finally, the gleno-humeral joint capsule was removed from the bone. The mediolateral length and anteroposterior width of the loose and firm attachment areas of the supraspinatus subregions and infraspinatus partitions to the capsule and those of the footprint areas of the supraspinatus and infraspinatus tendons and the capsule were roughly measured using a digital calliper (DC-10; Topman Co., Ltd, Miki, Japan). The measurements for footprint areas were performed based on previous reports [13, 15]. The measurement methods are shown in Fig. 2.

For histological assessment, we obtained coronal sections parallel to the tendons of the anterior region and posterior region of the supraspinatus according to Roh et al. and Kim et al. [10, 17] and sections of the superior, middle, and inferior partitions of the infraspinatus according to Fabrizio and Clemente as well as Bacle et al. [1, 7]. These samples were stained with Masson’s trichrome.

This study was approved by the Ethics Committee of Yamagata University School of Medicine (No. 315) in Yamagata, Japan as well as the Ethics Review Board of the Yamagata Prefectural University of Health Sciences (#1701-22), Yamagata, Japan.

SPSS statistical software (version 24.0; SPSS, Chicago, IL, USA) was used. To compare the areas of loose and firm attachments of the supraspinatus subregions and infraspinatus partitions, we performed a Kruskal–Wallis test followed by a Bonferroni post hoc test. Statistical significance was defined as a *p* value less than .05.

## Results

### Macroscopic morphology

In the supraspinatus, the anterior region muscle fibres connected to the thick anterior tendon fibres, and the posterior region muscle fibres connected to the thin posterior tendon fibres. From an inferior view, the muscle fibres of the anterior-deep and posterior-deep subregions defined by Kim et al. [10] were observed in agreement with the muscle fibre directions, and more than half of the deep muscle fibres of the anterior-deep subregion overlaid those of the posterior-deep subregion. The deep muscle and tendon fibres of the anterior-deep and posterior-deep subregions formed loose and firm attachments. In the infraspinatus, the tendon fibres of the superior, middle, and inferior partitions formed the conjoint tendon. The muscle fibres of the superior, middle, and inferior partitions according to Fabrizio and Clemente as well as to Bacle et al. [1, 7] were identified in agreement with muscle fibre directions. A portion of the deep muscle fibres of the inferior partition overlaid those of the middle partition. The deep muscle and tendon fibres of the middle partition and the remaining fibres of the inferior partition formed loose and firm attachments. The loose and firm attachment areas of inferior partition were small. Because the superior- partition muscle and tendon traversed over the middle partition muscle and tendon, the superior partition did not have any capsular attachments. Figure 1 shows the representative loose and firm attachments between the under surface of the supraspinatus subregions and infraspinatus partitions and the capsule (Fig. 1a–c). Figure 2 shows superior schematic views of the deeper surface of subregions of the anterior-deep subregion, posterior-deep subregion and middle partition that attached the capsule. Table 1 shows the stable configuration of loose and firm attachment sites in the posterior-deep subregion and middle partition compared to the variable patterns of those in the anterior-deep subregion and inferior partition (Table 1).

**Table 1:** Loose and firm capsular attachment sites to the capsule

Specimen	AD		PD		MP		IP	
	Loose attachment	Firm attachment	Loose attachment	Firm attachment	Loose attachment	Firm attachment	Loose attachment	Firm attachment
1	Muscle	Muscle and tendon	Muscle	Muscle and tendon	Muscle	Tendon	None	Muscle and tendon
2	Muscle	Tendon	Muscle	Muscle and tendon	Muscle	Tendon	None	Muscle and tendon
3	Muscle	Tendon	Muscle	Muscle and tendon	Muscle	Tendon	None	Muscle and tendon
4	Muscle	Tendon	Muscle	Muscle and tendon	Muscle	Tendon	None	Muscle and tendon
5	Muscle	Tendon	Muscle	Muscle and tendon	Muscle	Tendon	Muscle	Tendon
6	Muscle	Tendon	Muscle	Tendon	Muscle	Tendon	None	Muscle and tendon
7	Muscle	Tendon	Muscle	muscle and tendon	Muscle	Tendon	Muscle	Tendon
8	Muscle	Tendon	Muscle	muscle and tendon	Muscle	Tendon	None	Tendon
9	Muscle	Tendon	Muscle	muscle and tendon	Muscle	Tendon	None	Tendon
10	Tendon	Tendon	Muscle	muscle and tendon	Muscle	Tendon	Muscle	Tendon
11	Tendon	Tendon	Muscle	muscle and tendon	Muscle	Tendon	None	Muscle and tendon
12	Muscle	Tendon	Muscle	muscle and tendon	Muscle	Tendon	Muscle	Tendon
13	Muscle	Muscle and tendon	Muscle	muscle and tendon	Muscle	Tendon	Muscle	Tendon
Numbers which muscle attached to the capsule	*n* = 11	*n* = 2	*n* = 13	*n* = 12	*n* = 13	*n* = 0	*n* = 5	*n* = 6

The capsular attachment sites were stable in the PD and MP. In contrast, the capsular attachment sites of both the AD and IP were varied

*AD* anterior-deep subregion, *IP* inferior partition, *MP* middle partition, *PD* posterior-deep subregion

The measurement methods for the mediolateral lengths and anteroposterior widths of the loose and firm attachments of the anterior-deep subregion, posterior-deep subregion and middle partition are shown in Fig. 2, and Table 2 shows their mean values. Using the mediolateral and anteroposterior dimensions, the rectangular areas of loose and firm attachments of the anterior-deep subregion, posterior-deep subregion and middle partition were calculated. The mean rectangular areas of the loose attachment of the anterior-deep subregion, posterior-deep subregion and middle partition were 125.2 ± 80.7 mm^2^, 286.9 ± 147.6 mm^2^, and 658.7 ± 228.8 mm^2^, respectively. The loose attachment area of the middle partition was significantly greater than that of the anterior-deep and posterior-deep subregions, respectively (*p* < .001 and *p* = .006). There was no significant difference between the loose attachment areas of the anterior-deep and posterior-deep subregions (*p* = .058). The mean rectangular areas of the firm attachment of the anterior-deep subregion, posterior-deep subregion and middle partition were 118.8 ± 59.8 mm^2^, 267.8 ± 160.2 mm^2^ and 399.3 ± 176.7 mm^2^, respectively. Both firm attachment areas of the posterior-deep subregion and middle partition were significantly greater than those of the anterior-deep subregion (*p* = .013 and *p* = .000), while there was no significant difference between those of the posterior-deep subregion and middle partition (*p* = .181).

**Table 2:** Measurement of the capsular attachment and footprint: averages in millimetres

Capsular attachment											Footprint										
Mediolateral					Anteroposterior			Area			Mediolateral					Anteroposterior			Area		
Loose attachment								Loose attachment													
AD		PD		MP				AD	PD	MP											
16.1		18.5		28.1				125.2	286.9	658.7											
					AD	PD	MP				SSP	ISP	C1	C3	C5	SSP	ISP	Capsule	SSP	ISP	Capsule
					7.5	15.0	23.5				8.1	12.2	4.6	3.7	7.4	17.0	26.6	34.2	69.6	325.4	206.0
Firm attachment								Firm attachment													
AD	PD		MP					AD	PD	MP											
15.6	17.3		16.3					118.8	267.8	399.3											

*AD* anterior-deep subregion, *MP* middle partition, *PD* posterior-deep subregion, *SSP* supraspinatus tendon, *ISP* infraspinatus tendon, *C1* mediolateral length of the footprint of the capsule at the anterior margin of greater tubercle, *C3* mediolateral length of the footprint of the capsule at the posterior margin of the SSP tendon, *C5* mediolateral length of the footprint of the capsule at the posterior margin of ISP tendon

The footprint areas of the supraspinatus and infraspinatus tendons and of the capsule were triangular, parallelogrammic, and trapezoidal in shape, respectively. The footprint areas were calculated as triangles, parallelograms and trapezoids (using C1 and C5 for the mediolateral length), and their mean areas were 69.6 ± 18.4 mm^2^, 325.4 ± 70.7 mm^2^, and 206.0 ± 50.7 mm^2^, respectively (Table 2).

In an arch-like fashion, the transverse fibres remained at both the deeper surface of the rotator cuff tendon and the superficial surface of the capsule and just lateral to the medial margin of the firm attachment when the firm attachment was sharply separated at the level between the tendon and capsule (Fig. 1 c).

### Microscopic morphology

**Fig. 3:**
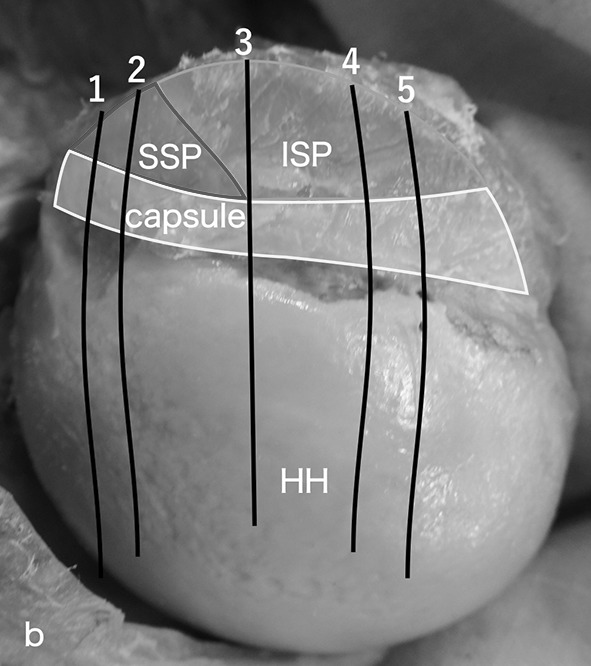
Orientations of the coronal section for microscopic observation. Lines 1–5 show coronal sections of the most anterior area of the anterior region tendon of the supraspinatus, of the posterior region tendon of the supraspinatus, of the superior partition tendon of the infraspinatus, of the middle partition tendon of the infraspinatus and of the inferior partition tendon of the infraspinatus, respectively. *HH* humeral head, *ISP* infraspinatus, *SSP* supraspinatus

**Fig. 4:**
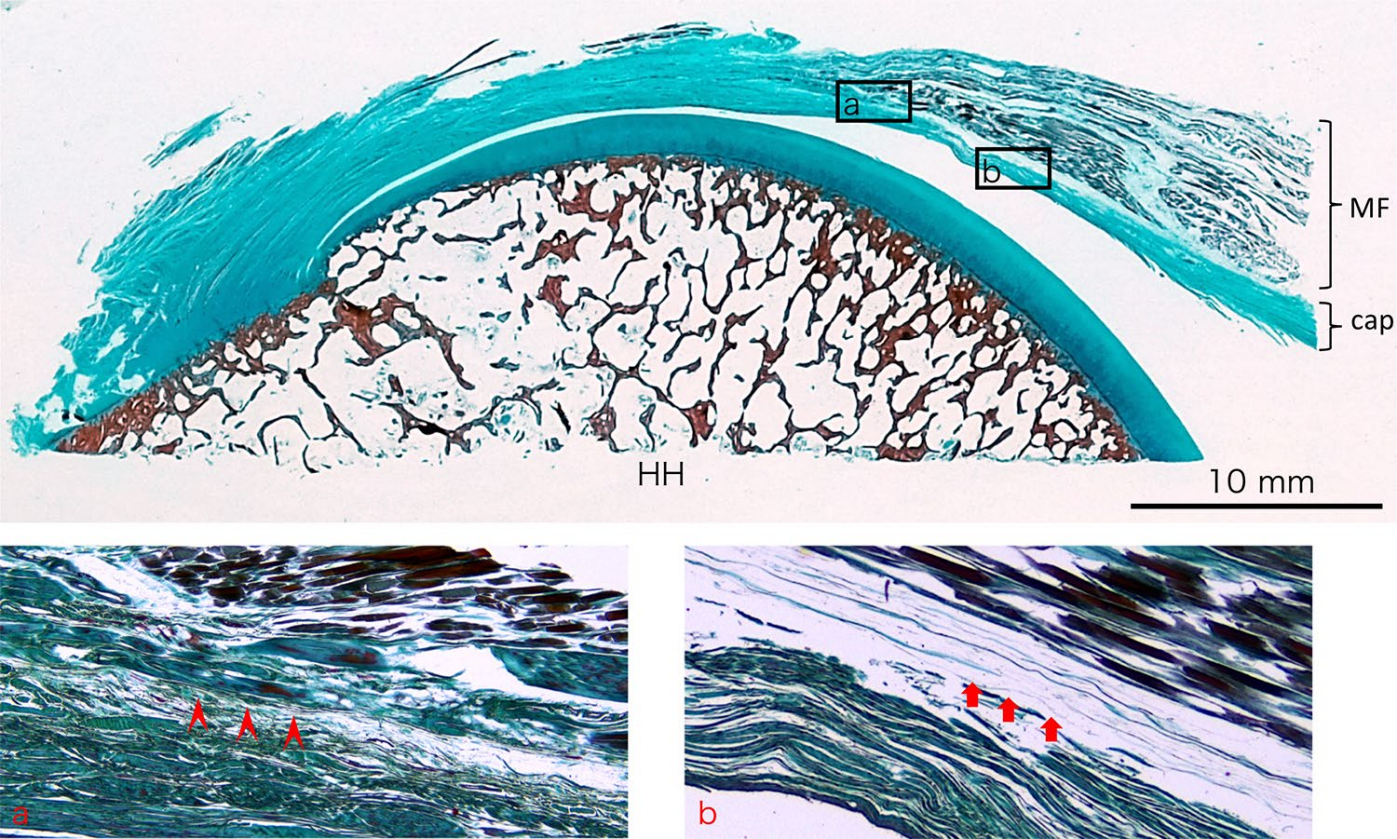
Coronal section of the posterior region tendon of the supraspinatus muscle. **a** Magnification of the square area of Fig. 4a. Lateral two-thirds of the capsule where the transverse fibres (arrowheads) were included were indistinguishable from the tendon and muscle fibres of the posterior-deep subregion because these fibres intermingled with the capsule, namely the firm attachment (open arrows). The transverse fibres (arrowheads) are located just lateral to the medial margin of the firm attachment. **b** Magnification of the square area of Fig. 3b. The medial one-third of the capsule was distinct from the deep muscle fibres because there were broader spaces and less dense connective tissues, namely, the loose attachment (closed arrows). *cap* capsule, *MF* muscle fibres, *HH* humeral head, *SSP* supraspinatus

**Fig. 5:**
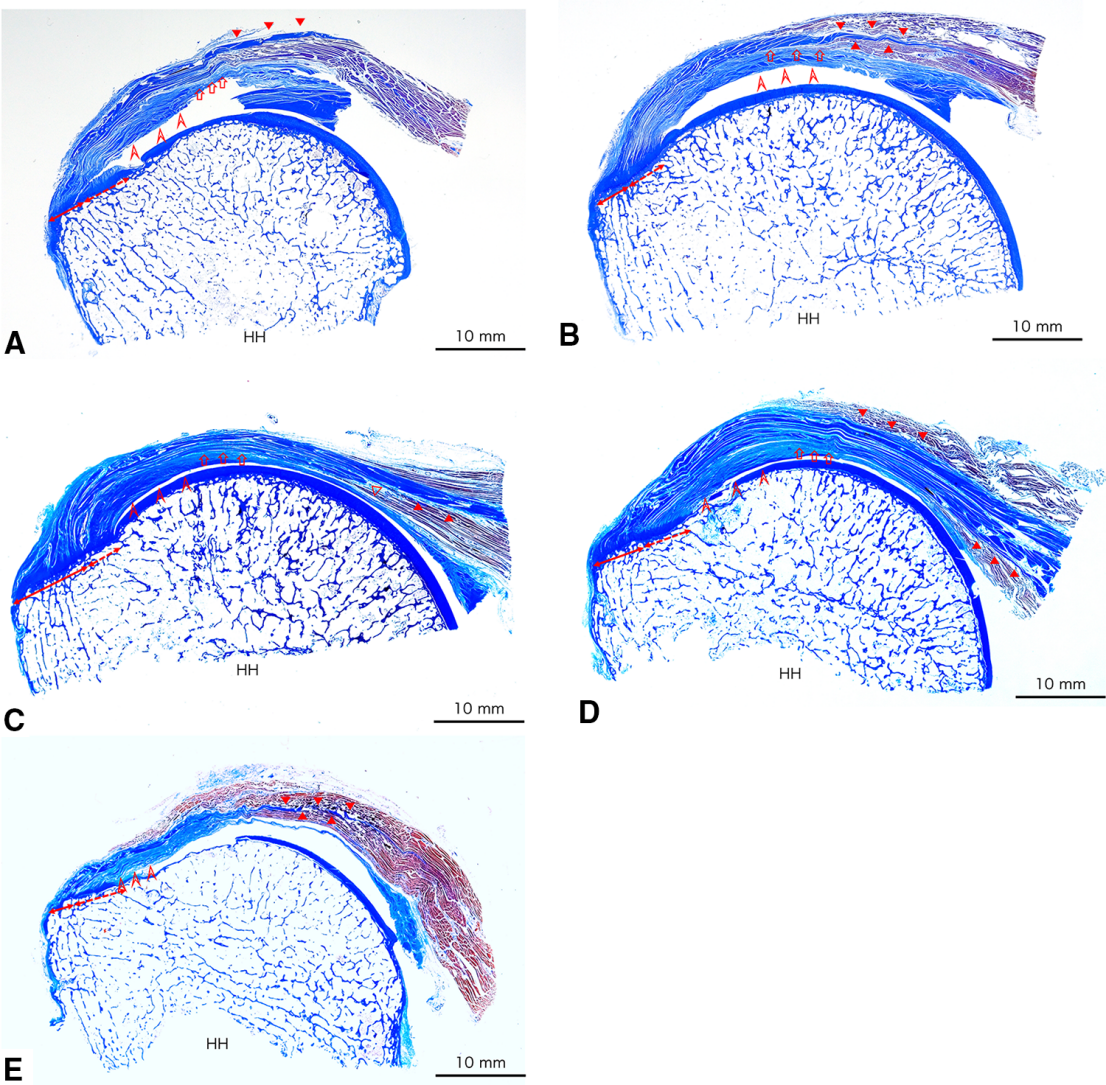
**a** Coronal section of the most anterior of the anterior region tendon of the supraspinatus muscle. The tendon fibres arose mainly from the superficial surface of the muscle belly (closed triangles) and traversed laterally. The tendon fibres and the capsule inserted to the lateral one-third of the superior facet (double head allows) and the medial two-thirds of the superior facet (dashed double head allows) through the attachment fibrocartilage, respectively. The relationship between the muscle belly and the capsule was unclear in this section because a medial part of the capsule was deficit. The capsule where the transverse fibres (open arrowheads) were included had the firm attachment area. **b** Coronal section of the posterior region tendon of the supraspinatus muscle. In this sample, the tendon fibres arose as an internal tendon between the posterior-deep subregion and the superficial subregion muscle belly (between closed triangles). The tendon fibres and capsule inserted into the lateral half of the superior facet (double head allows) and the medial half of the superior facet (dashed double head allows) through the attachment fibrocartilage, respectively. The lateral two-thirds of the capsule where the transverse fibres (open arrowheads) were included had the firm attachment area. In contrast, the medial one-third of the capsule formed the loose attachment. The thickness of the capsule was almost the same from medial to lateral. **c** Coronal section of the superior partition tendon of the infraspinatus muscle. The superior partition has a deep tendon (closed triangles) under the muscle belly, and the superficial aspect of the superior partition was covered with thin fascia. In this section, between the deep tendon of superior partition and the capsule, the cranial region of the middle partition tendon, which arose as an internal tendon (open triangle), had already appeared. The deep tendon of the superior partition and the cranial region of the middle partition tendon ran laterally. These tendon fibres and the capsule inserted to the lateral three-fourths of the middle facet (double head allows) and the medial one-fourth of the middle facet (dashed double head allows) through the attachment fibrocartilage, respectively. The lateral half of the capsule where the transverse fibres (arrowheads) were included had the firm attachment area. In contrast, the medial half of the capsule formed the loose attachment. The lateral half of the capsule was approximately two times thicker than the medial half of the capsule. **d** Coronal section of the middle partition tendon of the infraspinatus muscle. The caudal region of the middle partition tendon (between closed triangles) was identified as an internal tendon between the middle partition muscle fibres. The superficial surface of the middle partition was covered with thin fascia. The caudal region of the middle partition tendon and capsule inserted into the lateral half of the middle facet (double head allows) and the medial half of the facet (dashed double head allows) through the attachment fibrocartilage, respectively. The lateral half of the capsule where the transverse fibres (open arrowheads) were included had the firm attachment area. In contrast, the medial half of the capsule formed the loose attachment. The lateral half of the capsule became thicker than the medial half of the capsule. **e** Coronal section of the inferior partition tendon of the infraspinatus muscle. The inferior partition had a thin internal tendon (between red arrowheads). The tendon fibres and the capsule inserted into the lateral one-third of the middle facet (double head allows) and the medial two-thirds of the middle facet (dashed double head allows) through the attachment fibrocartilage, respectively. The lateral one-third of the capsule where the transverse fibres (open arrowheads) were included had the firm attachment area. In contrast, there were no loose or firm attachments between the medial two-thirds of the capsule and the inferior partition muscle and/or tendon. The lateral one-third became thicker than the medial two-thirds of the capsule. *HH* humeral head (colour figure online)

Figure 3 shows where the samples for histological observations were harvested (Fig. 3, lines 1–5). The lateral region of the gleno-humeral joint capsule, including the transverse fibres, was indistinguishable from the deep muscle and/or tendon fibres due to dense connections, whereas the medial region was distinct from the deep muscle fibres because there were broader spaces and less dense connective tissues (Fig. 4). These firm attachments were observed in the lateral two-thirds or lateral half of the capsule under the posterior region and middle partition (Fig. 5b–d), whereas the firm attachment was observed in the lateral one-third of the capsule under the inferior partition (Fig. 5e).

The tendon fibres joined some muscle fibres. The capsule was composed of less uniform fibres, including the transverse fibres, which were as deeply stained as the tendon fibres. Both the tendon fibres and the capsule inserted into the superior and middle facets through the attachment fibrocartilage. The attachment fibrocartilage was as deeply stained as the articular cartilage of the humeral head. The attachment fibrocartilage of the posterior region and middle partition was thicker than that of the inferior partition (Fig. 5b–e).

The tendon and capsule insertion length of the coronal section along with the most anterior section of the anterior region tendon of supraspinatus (Fig. 3, line 1), the posterior region tendon of the supraspinatus (Fig. 3, line 2), the superior partition tendon of the infraspinatus (Fig. 3, line 3), the middle partition tendon of the infraspinatus (Fig. 3, line 4) and the inferior partition tendon of the infraspinatus (Fig. 3, line 5) were the lateral one-third and medial two-thirds of the superior facet, lateral half and medial half of the superior facet, lateral three-fourths and one-fourth of the middle facet, lateral half and medial half of the middle facet and lateral one-third and medial two-thirds of the middle facet, respectively.

The lateral region of the capsule, which firmly attached the fibres of the muscle and/or tendon of subregions and included the transverse fibres, was thicker than the medial region (Fig. 5a–e). The transverse fibres were located just lateral to the medial edge of the firm attachment area. The transverse fibres were mostly medial in coronal sections of the posterior region and second most medial in those of the superior partition; additionally, the transverse fibres gradually shifted laterally while traversing inferiorly in an arch-like fashion (Fig. 5a–e).

## Discussion

The most important finding of this study is that the posterior-deep subregion of the supraspinatus and middle partition of the infraspinatus evenly occupied the capsular attachment area. The transverse fibres were located just lateral to the medial edge of the firm attachment area, and the thick capsule had a substantial footprint. Both tendon fibres and the capsule inserted into superior and middle facets through the attachment fibrocartilage.

Previously, Clark et al. found loose and firm attachments between the gleno-humeral joint capsule and the deeper surface of the rotator cuff [5]. In the present study, these capsular attachments were observed between the capsule and the deeper surface of the subregions of the anterior-deep and posterior-deep subregions of the supraspinatus and the middle and inferior partitions of the infraspinatus. Our macroscopic measurement showed that the middle partition comprised the significantly greatest area of the loose attachment and that the posterior-deep subregion and middle partition evenly occupied the firm attachment area. In contrast, the loose and firm attachment areas of the anterior-deep subregion were the smallest, and the attachments of the inferior partition were also very small. In addition, 92% of the shoulders formed loose and firm attachments with the deep muscle fibres of the posterior-deep subregion, and all shoulders formed loose and firm attachments with the deep muscle and tendon fibres of the middle partition, respectively. These capsular attachment sites were stable configurations. In contrast, the capsular attachment sites of both the anterior-deep subregion and inferior partition were unstable. When the muscle fibres are connected directly to the capsule, the subregion independently distributes its tension to the capsule. When the tendon fibres form a firm attachment with the capsule, the tension of the whole muscle may be transmitted to the capsule. In cases with various capsular attachment patterns, the tension distribution is unsettled. Microscopically, the firm attachment of the posterior-deep subregion and middle partition were longer than those of the inferior partition, and both tendon fibres of the posterior-deep subregion and middle partition and the capsule that firmly attached to them inserted into superior and middle facets through thicker attachment fibrocartilage, whereas the inferior partition tendon inserted into the middle facet with thinner attachment fibrocartilage. Therefore, the posterior-deep subregion and middle partition were essential parts of the capsular attachment since their capsular attachment areas were greater and capsular attachment patterns were settled, as well as both tendon fibres and capsule inserted into superior and middle facets through thicker attachment fibrocartilage. In contrast, the anterior-deep subregion and inferior partition seemed less important because their capsular attachment areas were smaller and capsular attachment patterns were variable, as well as both tendon fibres of the inferior partition and capsule inserted into the middle facet with thinner attachment fibrocartilage.

The capsular footprint occupied 34% of the superior and middle facets, while the footprint of the supraspinatus and infraspinatus tendons occupied 66% of them. The ratios of the mediolateral footprint length of the tendon to that of the capsule in microscopic observations were 1 to 2 in the coronal section of the most anterior section of the anterior region, 1 to 1 in that of the posterior region, 1 to 0.33 in that of the superior partition, 1 to 1 in that of the middle partition, and 1 to 3 in that of the inferior partition, while those in macroscopic measurements were 1 to 0.57 in the anterior margin of the supraspinatus, 1 to 0.30 in the posterior margin of the supraspinatus and 1 to 0.61 in the posterior margin of the infraspinatus. These discrepancies may depend on the difference in measurement manner: the macroscopic footprint was measured as the area beyond the facets where the tendon fibres continued to the periosteum, while the microscopic measurements were performed within the limits of the facets. The thick capsular footprint may be less negligible than it was previously thought to be, which is consistent with the findings by Mochizuki et al. and Nimura et al. [13, 15]. Microscopically, the insertions of the tendon fibres and thick capsule are essentially the same; that is, they are both inserted into the superior and middle facets through the thick attachment fibrocartilage. Benjamin et al. showed that the insertion of the supraspinatus was composed of the tendon, uncalcified fibrocartilage, calcified fibrocartilage and bone [2]. Fallon et al. called the uncalcified fibrocartilage the attachment fibrocartilage and suggested that attachment fibrocartilage of the rotator cuff muscle may function to resist compression or disburse the stress in the region of the tendon insertion into the greater tubercle [8]. Therefore, these similar entheses of the tendon fibres and the capsule suggest that the thick capsule may play an important functional role in a similar manner for tendon fibres.

Burkhart et al. previously identified the transverse fibres that extend anteriorly to the biceps and posteriorly to the inferior border of the infraspinatus in an arch-like fashion and called it a rotator cable [4]. In this study, we found transverse fibres corresponding to the rotator cable in both macroscopic and microscopic observations. These fibres were located just lateral to the medial edge of the firm attachment area in an arch-like fashion. As described above, the posterior-deep subregion and middle partition evenly occupied the firm attachment area, and they were the essential parts in capsular attachment. Looking down upon the humeral head, the posterior-deep subregion and middle partition fibres make an angle of approximately 90° and look like reins, including the transverse fibres regarded as a bit for the reins.

Burkhart et al. hypothesized that the rotator cable has a stress transfer function that transmitted the tensions generated by the rotator cuff into the humerus through each end of the cable’s span and called it the suspension bridge theory [3]. Clark et al. suggested that the loose attachment retracts redundant capsule tissue and that the firm attachment distributes some of the tension generated by the rotator cuff muscles into the capsule [5]. Yuri et al. found that the posterior-deep subregion of the supraspinatus had an independent function in the supraspinatus subregions and acted from 0° to 70° of external rotation [18]. Kuwahara et al. reported that the contractile force of the posterior-deep subregion and middle partition similarly increased with increasing external rotation angle [11]. Consequently, given that these subregions have distinct functions, each of them may spatio-temporally regulate the upward force of the humeral head via the firm attachments by pulling the rein of the posterior-deep subregion during abduction and drawing in the reins of the posterior-deep subregion and middle partition during external rotation.

Mochizuki et al. [14] demonstrated the efficacy of the independent repair technique of the capsule and the superficial layer of the tendon to the delaminated tear. One of the advantages of this method is the induction of concavity force of the humeral head by the capsule. Regarding the clinical relevance of this study, the repair of the capsule results in the repair of the firm attachment of posterior-deep subregion and middle partition because their deeper surface firmly attaches the capsule. The technique proposed by Mochizuki is an anatomical repair of all subregions of the supraspinatus and infraspinatus and the capsular attachment.

The limitation of this study was the small number of samples for microscopic observations.

## Conclusion

The posterior-deep subregion of the supraspinatus and middle partition of the infraspinatus evenly occupied the capsular attachment area. The transverse fibres were located just lateral to the medial edge of the firm attachment area, and the thick capsule had a substantial footprint. Both tendon fibres and the capsule inserted into the superior and middle facets through the attachment fibrocartilage.
